# Meclizine Inhibits Mitochondrial Respiration through Direct Targeting of Cytosolic Phosphoethanolamine Metabolism*[Fn FN2]

**DOI:** 10.1074/jbc.M113.489237

**Published:** 2013-10-19

**Authors:** Vishal M. Gohil, Lin Zhu, Charli D. Baker, Valentin Cracan, Abbas Yaseen, Mohit Jain, Clary B. Clish, Paul S. Brookes, Marica Bakovic, Vamsi K. Mootha

**Affiliations:** From the ‡ Departments of Molecular Biology and Medicine, Center for Human Genetic Research, Massachusetts General Hospital, Boston, Massachusetts 02114,; § Broad Institute, Cambridge, Massachusetts 02142,; ¶ Department of Systems Biology, Harvard Medical School, Boston, Massachusetts 02115,; ‖ Department of Biochemistry and Biophysics, Texas A&M University, College Station, Texas 77843,; ** Department of Human Health and Nutritional Sciences, University of Guelph, Guelph, Ontario NIG 2W1, Canada, and; ‡‡ Department of Anesthesiology, University of Rochester Medical Center, Rochester, New York 14642

**Keywords:** Energy Metabolism, Metabolomics, Mitochondria, Phosphatidylethanolamine, Respiration, Meclizine, Phosphoethanolamine

## Abstract

We recently identified meclizine, an over-the-counter drug, as an inhibitor of mitochondrial respiration. Curiously, meclizine blunted respiration in intact cells but not in isolated mitochondria, suggesting an unorthodox mechanism. Using a metabolic profiling approach, we now show that treatment with meclizine leads to a sharp elevation of cellular phosphoethanolamine, an intermediate in the ethanolamine branch of the Kennedy pathway of phosphatidylethanolamine biosynthesis. Metabolic labeling and *in vitro* enzyme assays confirmed direct inhibition of the cytosolic enzyme CTP:phosphoethanolamine cytidylyltransferase (PCYT2). Inhibition of PCYT2 by meclizine led to rapid accumulation of its substrate, phosphoethanolamine, which is itself an inhibitor of mitochondrial respiration. Our work identifies the first pharmacologic inhibitor of the Kennedy pathway, demonstrates that its biosynthetic intermediate is an endogenous inhibitor of respiration, and provides key mechanistic insights that may facilitate repurposing meclizine for disorders of energy metabolism.

## Teaser

**Background:** Previous studies have shown that meclizine inhibits respiration in intact cells, but not in isolated mitochondria, via an unknown mechanism.

**Results:** Meclizine directly inhibits PCYT2 (CTP:phosphoethanolamine cytidylyltransferase).

**Conclusion:** Meclizine attenuates mitochondrial respiration by directly inhibiting the Kennedy pathway of phosphatidylethanolamine biosynthesis.

**Significance:** We identified a novel molecular target of meclizine, an over-the-counter antinausea drug, raising possibilities for new clinical applications.

## Introduction

Although mitochondrial respiration is crucial for cellular energetics and redox balance, during certain pathological conditions, respiration can actually contribute to pathogenesis (1). Attenuation of mitochondrial respiration has been proposed as a therapeutic strategy in a number of human disorders, including ischemia-reperfusion injury, neurodegeneration, autoimmune disease, and cancer (2). Many naturally occurring as well as synthetic compounds targeting the mitochondrial respiratory chain are available, but their clinical utility is limited by their narrow therapeutic index (3); thus, there is an unmet need for discovering new classes of drugs that can safely modulate mitochondrial respiration.

We recently identified meclizine, an over-the-counter anti-nausea drug, in a “nutrient-sensitized” chemical screen aimed at identifying compounds that attenuate mitochondrial respiration (4). *In vivo* follow-up studies demonstrated that meclizine could be protective against heart attack (4), stroke (4), and neurodegeneration (5) in animal models. Meclizine is a first generation piperazine class of H_1_-antihistamine that has been in use for decades for prophylaxis against nausea and vertigo (6). Like many H_1_-antihistamines, meclizine has anticholinergic activity (7), and it has also been shown to target constitutive androstane receptors (8).

Meclizine inhibition of respiration had not been reported before, and unlike classical respiratory inhibitors (*e.g.* antimycin and rotenone), meclizine did not inhibit respiration in isolated mitochondria but rather only in intact cells (4). We previously reported that this effect was independent of its antihistaminergic and anticholinergic activity (4), but the precise mechanism remained unknown. Curiously, the kinetics of meclizine-mediated inhibition of respiration were on the time scale of minutes (4), which is far too fast for a transcriptional mechanism but slower than direct inhibitors of the respiratory chain, suggesting that the inhibition arose from a potentially novel mechanism perhaps through intracellular accumulation of a meclizine-derived active metabolite or by perturbing metabolism.

To gain insights into the mechanism of meclizine action, we performed global metabolic profiling of meclizine-treated cells to detect alterations in intracellular metabolites of intermediary metabolism. Metabolic profiling revealed a sharp increase in intracellular levels of phosphoethanolamine (PEtn),^5^ an intermediate in the CDP-ethanolamine (Etn) Kennedy pathway of phosphatidylethanolamine (PE) biosynthesis. Follow-up biochemical experiments confirmed the direct inhibition of CTP:phosphoethanolamine cytidylyltransferase (PCYT2), a rate-limiting enzyme of the CDP-Etn Kennedy pathway. The inhibition of PCYT2 results in the buildup of its substrate, PEtn, which itself directly inhibits mitochondrial respiration. Our work thus identifies a novel molecular target of meclizine and links the CDP-Etn Kennedy pathway to mitochondrial respiration.

## EXPERIMENTAL PROCEDURES

### 

#### 

##### Metabolite Profiling

Metabolite profiling was performed on MCH58 fibroblasts following treatment with 50 μm meclizine or vehicle control for 5 h using methods similar to those described previously (9). Briefly, low passage MCH58 cells were cultured on 6-cm tissue culture dishes in 4 ml of culture medium to 90% confluence and a final yield of 1 × 10^6^ cells. For assessment of intracellular metabolites, medium was aspirated from the above tissue culture dishes, and cells were gently washed with 4 ml of phosphate-buffered saline (PBS) to ensure complete removal of residual medium metabolites. After removal of PBS, cellular metabolism was quenched with immediate addition of 1 ml of precooled (−80 °C) methanol extraction solution (80% methanol, 20% H_2_O). Cells were scraped in extraction solution, vortexed, and centrifuged, and the resulting supernatant was collected and stored at −80 °C. At the time of measurement, 100 μl of supernatant was diluted 1:1 with methanol extraction solution. The resulting solution was evaporated under nitrogen, the samples were reconstituted in 60 μl of high performance liquid chromatography (HPLC) grade water, and metabolites were assessed. Six biological replicates were assessed for each group. Analyses of endogenous metabolites were performed using a liquid chromatography-tandem mass spectrometry (LC-MS) system composed of a 4000 QTRAP triple quadrupole mass spectrometer (AB Sciex) coupled to three Agilent 1100 binary HPLC pumps (Agilent Technologies) and an HTS PAL autosampler (LEAP Technologies) equipped with three injection ports and a column selector valve. Three multiplexed chromatographic methods were configured for the analyses of each sample. LC method 1 used a Luna Phenyl-Hexyl column (Phenomenex) with a linear gradient of water/acetonitrile/acetic acid (initial proportions, 100:0:0.001; final proportions, 10:90:0.001). LC method 2 used a Luna NH_2_ column (Phenomenex) with a linear gradient using acetonitrile/water containing 0.25% ammonium hydroxide and 10 mm ammonium acetate (acetonitrile/water proportions were 80:20 at the beginning of the gradient and 20:80 at its conclusion). LC method 3 used a Synergi Polar-RP column (Phenomenex) and gradient elution with 5% acetonitrile, 5 mm ammonium acetate (mobile phase A) and 95% acetonitrile, 5 mm ammonium acetate (mobile phase B). MS data were acquired using multiple reaction monitoring in both the positive (LC method 1) and negative (LC methods 2 and 3) ion modes. During the development of this method, authentic reference compounds were used to determine LC retention times and to tune multiple reaction monitoring transitions. Metabolite quantification was performed by integrating peak areas for specific multiple reaction monitoring transitions using MultiQuant software (version 1.1; AB Sciex), and all integrated peaks were manually reviewed for quality. Extraction and quantification procedures were optimized prior to assessment of samples in this study to ensure measured intracellular and medium metabolites were within the linear range of detection. Confirmation of the PEtn peak was performed using an exogenous PEtn standard (Sigma P0503). The relative quantification of PEtn in MCH58 skin fibroblasts or mouse striatal cells (STHdh^Q7/7^) was also confirmed using hydrophilic interaction liquid chromatography (10).

##### Determination of the Intracellular Concentration of PEtn and Phosphocholine (PCho)

Intracellular concentrations of PEtn and PCho in meclizine-treated MCH58 fibroblasts and *PCYT2* knockdown cells were determined as follows. MCH58 fibroblast cells were seeded into a 6-well plate (0.2 × 10^6^ cells/well). After 20 h of growth, three wells were treated with 50 μm meclizine, and the three remaining wells were treated with DMSO for ∼5 h. Cells were scraped and collected in methanol extraction solution (80% methanol, 20% H_2_O), and PEtn and PCho levels were quantified by LC-MS using commercially available standards. Cell number and mean cellular diameter of MCH58 cells were determined using a Beckman Coulter Z-series cell counter in a parallel plate, which was subsequently used to calculate the intracellular concentration of PEtn and PCho.

##### Radiolabeling of Kennedy Pathway Intermediates

Radiolabeling of CDP-Etn Kennedy pathway intermediates was performed as described previously (11). Briefly, MCH58 fibroblasts were cultured in the presence of 50 μm meclizine for 5 h to 60% confluence at which point [^14^C]Etn (0.5 μCi/dish) was added, and cells were further incubated for 24 h. Lipids were extracted using the method of Bligh and Dyer (12). The total radioactivity from the water/methanol phase and that of the lipid phase were measured separately. [^14^C]PE was separated from the lipid phase using TLC in a solvent consisting of methanol/chloroform/ammonia (65:35:5). Etn, PEtn, and CDP-Etn were separated from the water/methanol phase using TLC in a solution of methanol, 0.5% NaCl, ammonia (50:50:5). The separated ^14^C-labeled compounds were measured by scintillation counting.

##### PCYT2 Enzyme Assay

PCYT2 activity was assayed as described previously with minor modifications (13). Briefly, a 50-μl mixture of 20 mm Tris-HCl buffer, pH 7.8, 10 mm MgCl_2_, 5 mm DTT, 650 μm CTP, 650 μm unlabeled PEtn, and 65 μm [^14^C]PEtn was incubated with 0.4 μg of purified PCYT2 at 37 °C for 15 min. Reactions were terminated by boiling for 2 min. Meclizine was added at the indicated concentration in the reaction mixture. Reaction mixtures were then loaded onto silica gel G plates with CDP-Etn and PEtn standards and separated in a solvent system of methanol, 0.5% NaCl, ammonia (50:50:5). Plates were then sprayed with 1% ninhydrin, and the CDP-Etn product was quantified by liquid scintillation counting.

##### Construction of PCYT2 Knockdown Cell Lines

The shRNA constructs targeting *PCYT2* were purchased from Open Biosystems, and the empty vector pLKO.1 was obtained from the Broad Institute (Cambridge, MA). Five independent shRNAs were used to construct MCH58 knockdown cell lines. We chose the two best knockdown cell lines to perform radiolabeling and bioenergetics assays. The shRNA sequences for the two best knockdowns are: kd1, 5′-CCCATCATGAATCTGCATGAA-3′; kd2, 5′-TCACGGCAAGACAGAAATTAT-3′. The lentiviral particles were produced in HEK293T cells as described previously (14). Infection of MCH58 fibroblasts with lentiviral particles, selection in puromycin (2 μg/ml), and their expansion to make stable knockdowns were performed essentially as described before (15). The *PCYT2* mRNA levels in knockdowns were quantified by quantitative RT-PCR using a TaqMan assay (Applied Biosystems).

##### Bioenergetic Assays

Oxygen consumption rate (OCR; a measure of mitochondrial respiration) and extracellular acidification rate (ECAR; a measure of glycolysis) measurements in intact cells were carried out as described previously (4, 15) with minor modifications. Briefly, MCH58 fibroblasts (control and *PCYT2* knockdowns) were seeded in XF24 24-well cell culture microplates (Seahorse Bioscience) at 30,000 cells/well in 25 mm glucose containing DMEM and incubated at 37 °C in 5% CO_2_ for ∼20 h. Prior to the measurements, the growth medium was replaced with ∼925 μl of assay medium (Seahorse Bioscience). The cells were incubated at 37 °C for 60 min in the assay medium prior to OCR/ECAR measurements. The measurements were performed simultaneously every 7 min after a 2-min mix and 2-min wait period. Three base-line OCR/ECAR measurements were recorded prior to the addition of 50 μm meclizine dihydrochloride (MP Biomedicals 155341). To test the effect of PEtn *in vitro*, mitochondria were isolated from C57BL/6 mouse kidneys as described previously (4). Simultaneous monitoring of oxygen consumption, membrane potential, and NADH and FAD^+^ fluorescence was performed using a custom-made spectrophotometer. The reaction mixture contained mitochondria (0.35 mg/ml) and tetramethylrhodamine methyl ester (0.5 μm) in 0.5 ml of buffer A (137 mm KCl, 10 mm HEPES, pH 7.4, 2.5 mm MgCl_2_, 0.1% BSA). During the experiment, glutamate/malate (4.6 mm), ADP (180 μm), and PEtn (0, 3.2, and 8 mm) were added. The following excitations wavelengths were used for NADH, FAD^+^, and tetramethylrhodamine methyl ester: 365, 470, and 530 nm. Data were acquired by integrating emission signals at 430–480, 520–560, and 580–600 nm for NADH, FAD^+^, and tetramethylrhodamine methyl ester, respectively. For traces showing oxygen consumption of purified mitochondria in the presence of PCho, 0.5 ml of buffer A contained kidney mitochondria (0.2 mg/ml), glutamate/malate (4.6 mm), ADP (200 μm), and PCho (0–8 mm). Because PCho was commercially available only as a Ca^2+^ salt, a 160 mm stock was prepared in 500 mm EGTA, H_2_O solution.

##### Determination of IC_50_ for PEtn Inhibition of Mitochondrial Oxygen Consumption

MitoXpress oxygen-sensitive probe (Luxcel Biosciences, Ireland) was used to monitor glutamate/malate-driven respiration of mouse kidney mitochondria in 96-well plate format (16). Each well contained glutamate/malate (11.5 mm), 20 μl of MitoExpress probe (dry probe was dissolved in 1 ml of water), mitochondria (0.5 mg/ml), and PEtn (0–50 mm) in 200 μl of total volume of buffer A. Two injections of ADP (487 μm final) were made using the injector of an EnVision 2104 plate reader (PerkinElmer Life Sciences). Time-resolved fluorescence was measured with the following settings: delay and gate times, 70 and 30 μs, respectively. Data analysis was performed using SigmaPlot 12.3. Briefly, slopes of basal respiration (state 2) and ADP-activated respiration (state 3) were taken, and the data were normalized against maximum respiration with no PEtn.

##### PE Determination

MCH58 fibroblasts were treated with 50 μm meclizine or DMSO for 5 h. Mitochondria were isolated from ∼1.5 g of cells (wet weight) using the Mitochondria Isolation kit for cultured cells (Abcam MS852). Total phospholipids were extracted from whole cells or isolated mitochondria (1.5 mg of mitochondrial protein) as described previously (17). Briefly, phospholipids were extracted in 3 ml of chloroform/methanol (2:1) by shaking for 1 h followed by addition of 600 μl of 0.9% NaCl solution and vortexing for an additional 15 min. The aqueous and organic layers were separated via centrifugation (300 × *g* for 5min) followed by an additional wash with 500 μl of H_2_O. The bottom layer (organic phase) was evaporated to dryness under N_2_ gas. The resulting lipid film was resuspended in 90 μl of 2:1 chloroform/methanol and separated on silica gel 60 TLC plates (EMD Millipore 1.05721.0001) using a solvent system consisting of chloroform/acetic acid/methanol/water (75:25:5:2.2). The TLC plates were then dried and exposed to iodine vapor to visualize phospholipid spots. The identity of each spot was verified using phospholipid standards (Sigma PH9-1KT). Individual spots were scraped, and phospholipid phosphorus was quantified using the method of Bartlett (18).

##### Testing the Combined Effect of Meclizine and Etn on MCH58 Fibroblast ATP Levels

MCH58 fibroblasts were seeded at 10,000 cells/well in a 96-well plate in high glucose DMEM and allowed to grow overnight. After ∼ 20 h, growth medium was replaced with 10 mm galactose medium containing different concentrations of meclizine (0, 12.5, 25, and 50 μm) and Etn (0, 0.2, 1, and 5 mm). The ATP levels in the cells were measured using CellTiter-Glo reagent (Promega) 24 h after the treatment.

## RESULTS

### 

#### 

##### Meclizine Treatment Results in the Elevation of PEtn across Multiple Mammalian Cell Types

**FIGURE 1.**
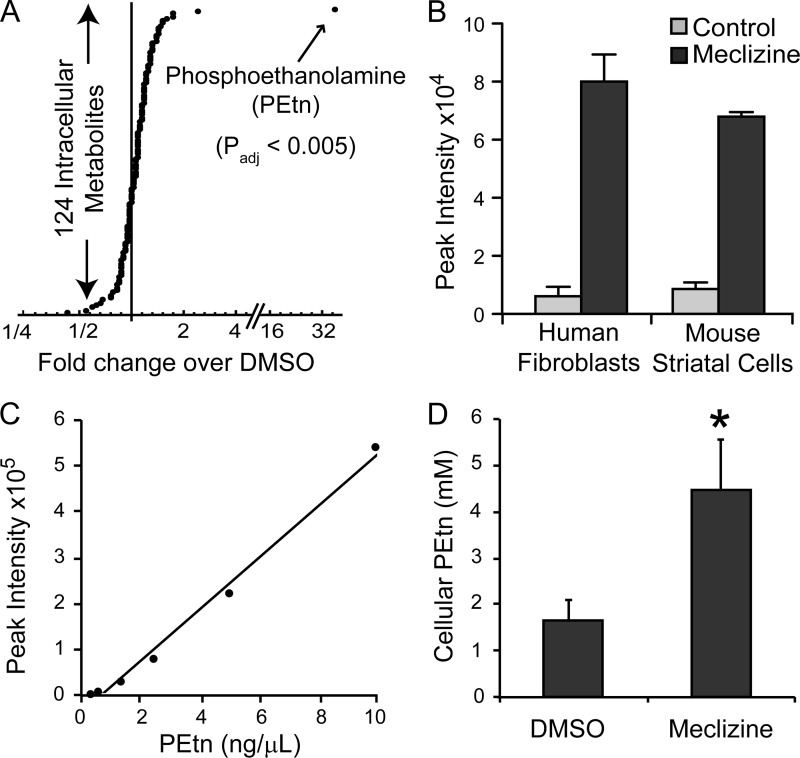
**Phosphoethanolamine is elevated in meclizine-treated cells.** *A*, mass spectrometry-based quantification of 124 intracellular metabolites from MCH58 human fibroblasts treated with 50 μm meclizine for 5 h. Each data point represents an average of six values for DMSO and meclizine-treated samples. *B*, hydrophilic interaction liquid chromatography-mass spectrometry-based relative quantification of PEtn from human fibroblasts (MCH58) or mouse striatal cells (STHdh^Q7/7^) treated with DMSO or 50 μm meclizine for 5 h. Data are expressed as mean ± S.D. (*n* = 6 for fibroblasts; *n* = 3 for striatal cells). *C*, the calibration curve used for the quantitative analysis of PEtn in meclizine-treated samples was obtained by LC-MS using PEtn standards. *D*, the cellular concentration of PEtn in MCH58 fibroblasts treated with meclizine (50 μm) or DMSO was determined by counting cell number, calculating total cellular volume using a Beckman Coulter Z-series cell counter, and measuring absolute PEtn levels by mass spectrometry. *Error bars* represent S.D. (*n* = 3). *, *p* < 0.05 as determined by *t* test.

To decipher the mechanism by which meclizine blunts respiration, we used a metabolomics approach because previous reports have shown that the technology can reveal targets of desired and undesired actions of drugs (19, 20). We performed mass spectrometry-based metabolic profiling of human immortalized skin fibroblast (MCH58) cells treated with 50 μm meclizine for 5 h. Our choice of MCH58 immortalized human skin fibroblasts was guided by our previous study characterizing the effect of meclizine on mitochondrial energy metabolism (4). Of the 124 metabolites that were measured reproducibly across six biological replicates, the most striking change corresponded to the PEtn signal, which increased nearly 35-fold (*p*_adj_ < 0.005), well outside the background distribution (Fig. 1*A* and supplemental Table 1). We further confirmed the identity and up-regulation of PEtn levels using hydrophilic interaction liquid chromatography-mass spectrometry in human fibroblasts and mouse striatal cells (Fig. 1*B*). These data are consistent with our previous observation showing that meclizine attenuates respiration across multiple cell types (4). Using PEtn standards, we quantified the absolute levels of PEtn in fibroblasts and showed that within 5 h of meclizine treatment the intracellular concentration of PEtn increased from ∼1.5 to 4 mm (Fig. 1, *C* and *D*).

##### The Pattern of CDP-Etn Kennedy Pathway Intermediates in Meclizine-treated Cells Is Consistent with PCYT2 Inhibition

**FIGURE 2.**
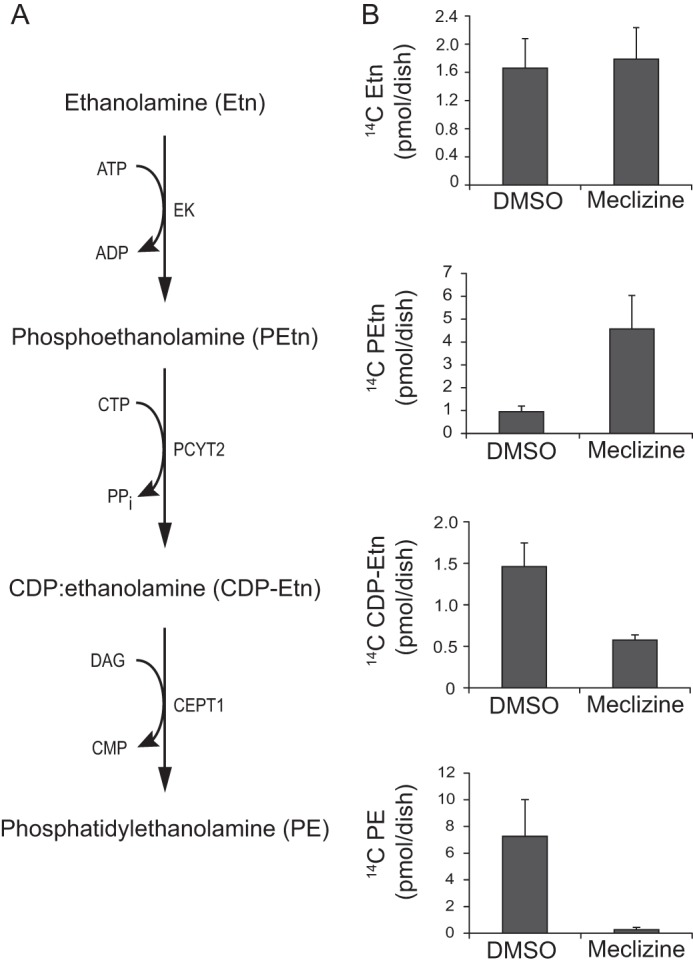
**Metabolic labeling of Kennedy pathway intermediates.** *A*, enzymatic reactions depicting synthesis of Kennedy pathway intermediates. *B*, quantification of radiolabeled Kennedy pathway intermediates in meclizine-treated MCH58 fibroblasts. MCH58 fibroblasts were cultured in the presence of 50 μm meclizine or DMSO for 5 h at which point [^14^C]Etn (0.5 μCi/dish) was added, and cells were further incubated for 24 h. The ^14^C-labeled compounds were separated as described under “Experimental Procedures” and measured by scintillation counting. *Error bars* represent S.D. (*n* = 5). *EK*, Etn kinase; *DAG*, diacylglycerol.

PEtn is an intermediate in the CDP-Etn Kennedy pathway of PE biosynthesis (21, 22). The mammalian CDP-Etn Kennedy pathway consists of three enzymatic steps. Etn is first phosphorylated by Etn kinase to PEtn, which is converted to CDP-Etn by PCYT2 (also referred to as ECT). Finally Etn phosphotransferase (CEPT1) catalyzes the CDP-Etn conversion to PE (Fig. 2*A*). To understand why PEtn levels rise with meclizine, vehicle and meclizine-treated cells were incubated with [^14^C]Etn for 24 h, and the incorporation of the radiolabel was measured in the intermediates of the CDP-Etn Kennedy pathway. With meclizine treatment, [^14^C]PEtn levels were increased, whereas radiolabel incorporation into downstream metabolites, CDP-Etn and PE, was decreased (Fig. 2*B*). These results raise the hypothesis that meclizine directly blocks PCYT2 enzymatic activity, which catalyzes the conversion of PEtn to CDP-Etn.

##### Meclizine Is a Non-competitive Inhibitor of PCYT2

**FIGURE 3.**
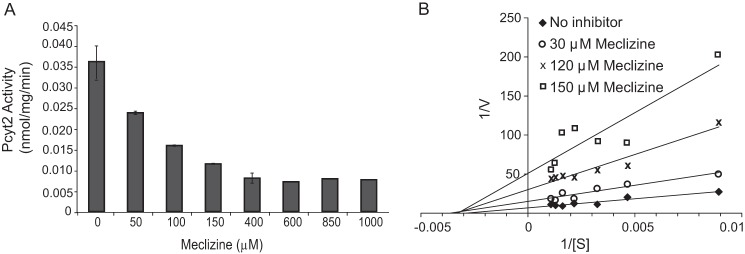
**Meclizine inhibits recombinant purified PCYT2.** *A*, *in vitro* activity of PCYT2 (0.4 μg) measured using 650 μm CTP, 650 μm PEtn, and 65 μm ^14^C-labeled PEtn (55 μCi/μmol) in the presence of varying meclizine concentrations. *B*, inhibition kinetics of PCYT2 with PEtn at a fixed concentration of 650 μm and 65 μm ^14^C-labeled PEtn (55 μCi/μmol) in the presence of varying concentrations of CTP (100–1000 μm) and meclizine (0–150 μm). All experiments were performed twice in duplicates. *Error bars* represent S.D.

To confirm whether meclizine directly inhibits PCYT2, we performed an *in vitro* enzyme assay with purified recombinant mouse PCYT2 (13) in the presence of varying concentrations of meclizine. Meclizine inhibited PCYT2 enzyme activity in a dose-dependent manner (Fig. 3*A*), and the Lineweaver-Burke plot of the PCYT2 enzyme kinetics suggests a non-competitive inhibition with an approximate *K_i_* of 31 μm (Fig. 3*B*).

##### PCYT2 Knockdown Cells Partially Mimic the Meclizine Effect

**FIGURE 4.**
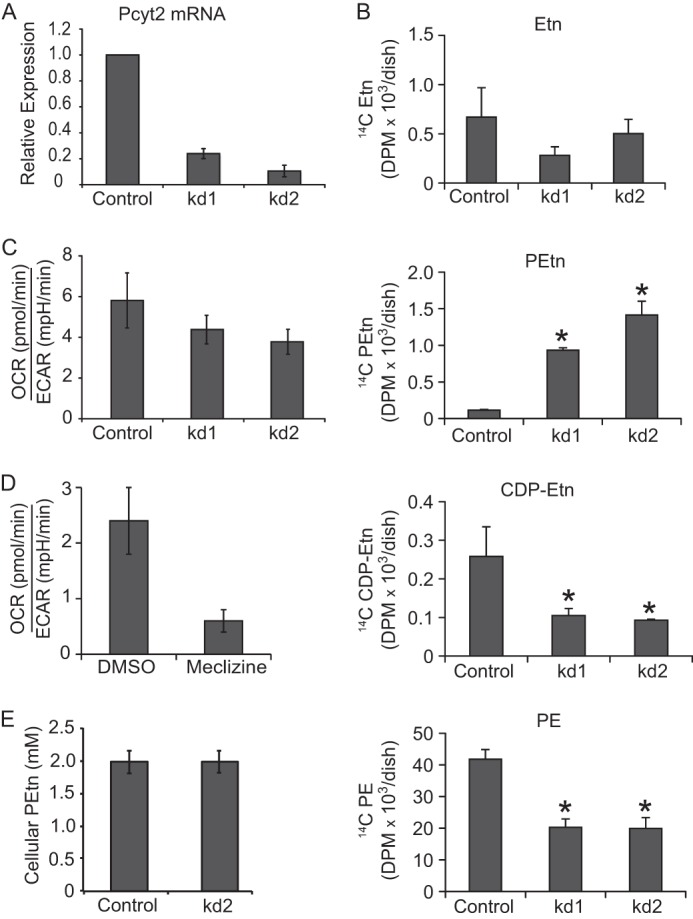
**Characterization of *PCYT2* knockdown cells.** *A*, *PCYT2* mRNA quantified by performing quantitative RT-PCR on RNA extracted from MCH58 fibroblasts infected with an empty vector (*Control*) or two independent shRNAs (*kd1* and *kd2*) targeting *PCYT2. B*, quantification of the Kennedy pathway intermediates in control and *PCYT2* knockdown fibroblasts. Experiments were done two times independently in triplicates (*, *p* < 0.05 as determined by *t* test). *C*, ratio of OCR, a measure of mitochondrial respiration, to ECAR, a measure of glycolysis, in control and *PCYT2* knockdown fibroblasts. *Error bars* represent S.D. (*n* ≥ 5). *D*, OCR to ECAR ratio in vehicle (DMSO)- or meclizine (50 μm)-treated fibroblasts. *Error bars* represent S.D. (*n* ≥ 4). *E*, the cellular concentration PEtn in control and *PCYT2* knockdown fibroblasts was determined by counting cell number, calculating total cellular volume using a Beckman Coulter Z-series cell counter, and measuring absolute PEtn levels by mass spectrometry. *Error bars* represent S.D. (*n* = 3). *mpH*, milli-pH units.

We next sought to phenocopy the effects of meclizine using RNAi against *PCYT2*. We stably silenced *PCYT2* in MCH58 human skin fibroblasts using five hairpins and focused on two hairpins that achieved 77 and 90% knockdown at the RNA level (Fig. 4*A*). We treated control and knockdown cells with [^14^C]Etn and quantified CDP-Etn Kennedy pathway intermediates. As expected, we observed an increase in [^14^C]PEtn levels and a decrease in downstream PCYT2 products CDP-[^14^C]Etn and [^14^C]PE, although the magnitude of changes was not as striking as with meclizine (Fig. 4*B*). The best knockdown of *PCYT2* showed a slight reduction in basal oxygen consumption and a corresponding increase in glycolysis (Fig. 4*C*), although the magnitude of effect was small when compared with meclizine-treated fibroblasts (Fig. 4*D*). This difference between chemical inhibition of PCYT2 by meclizine and genetic depletion of *PCYT2* by shRNA was consistent with our observation that a stable *PCYT2* knockdown is incomplete and does not result in the sustained accumulation of PEtn (Fig. 4*E*).

##### PEtn Inhibits Respiration in Suspensions of Isolated Mitochondria

**FIGURE 5.**
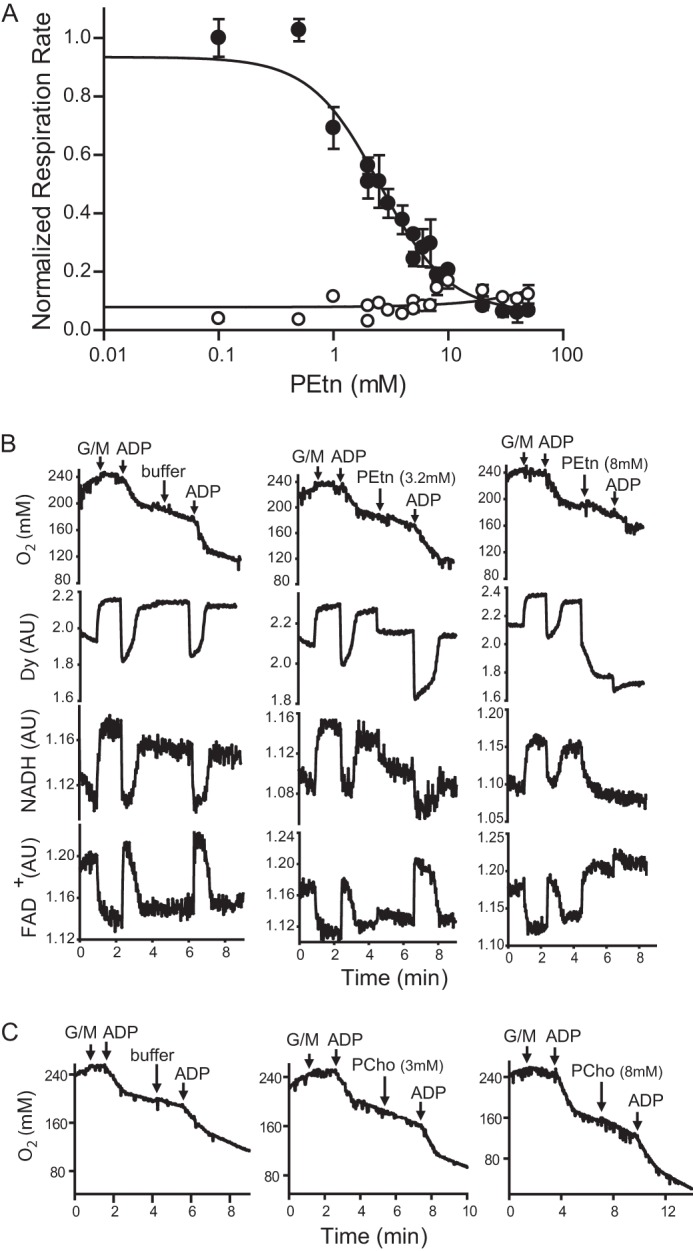
**Effects of phosphoethanolamine on mitochondrial bioenergetics.** *A*, the effect of increasing PEtn concentrations on glutamate/malate-driven ADP-activated respiration (●) and basal respiration (○) in isolated mouse kidney mitochondria. Rates of oxygen consumption are normalized to ADP-stimulated respiration in the absence of PEtn. Data are representative of three independent trials. *Error bars* represent deviation from a linear fit when corresponding slopes were analyzed. *B*, simultaneous measurements of oxygen consumption, membrane potential, and NADH and FAD^+^ fluorescence. Purified mouse kidney mitochondria (0.35 mg/ml) and tetramethylrhodamine methyl ester (0.5 μm) were incubated in buffer A. Glutamate/malate (*G/M*) (4.6 mm), ADP (180 μm), and PEtn (0, 3.2, and 8 mm) were added at the time points indicated by *arrows*. Traces are representative of at least three independent experiments. *C*, for traces showing oxygen consumption in the presence of PCho, the assay mixture contained mouse kidney mitochondria (0.2 mg/ml) in buffer A. The respiratory substrates glutamate/malate (*G/M*) (4.6 mm), ADP (200 μm), and PCho (0, 3, and 8 mm) were added at the time points indicated by *arrows*. Traces are representative of three independent experiments. *AU*, arbitrary units.

It is notable that a previous study showed that Etn and PEtn can directly inhibit respiration in isolated suspensions of mitochondria (23), raising the hypothesis that meclizine is blunting respiration *in vivo* via an elevation of PEtn. We found that PEtn did not impact basal (state 2) mitochondrial respiration but indeed had a strong inhibitory effect on ADP-stimulated (state 3) respiration (Fig. 5*A*) with an IC_50_ of 3.13 ± 0.54 mm. PEtn inhibited ADP-driven respiration either with glutamate/malate (Fig. 5, *A* and *B*) or with succinate/rotenone respiration (data now shown), suggesting that the inhibitory effect is not specific to respiratory complex I or complex II or to a specific fuel substrate. In assays for mitochondrial physiology, we found that the addition of PEtn led to decreased NADH and increased FAD^+^ intrinsic fluorescence together with a reduction of membrane potential (Fig. 5*B*). Notably, similar concentrations of PCho, an intermediate in the CDP-choline branch of the Kennedy pathway of phosphatidylcholine biosynthesis, did not inhibit respiration in suspensions of isolated mitochondria, indicating that the inhibitory effect of PEtn on mitochondrial respiration was specific (Fig. 5*C*). Moreover, we also measured PCho levels to determine whether meclizine-mediated elevation of PEtn is due to the specific inhibition of PCYT2. We observed a decrease in the cellular concentration of PCho from 6.14 ± 0.43 to 2.79 ± 0.37 mm with meclizine treatment, making PCho an unlikely contributor to the effect of meclizine on mitochondrial respiration *in vivo*.

##### Meclizine Treatment Does Not Alter Steady State Levels of Mitochondrial PE

**FIGURE 6.**
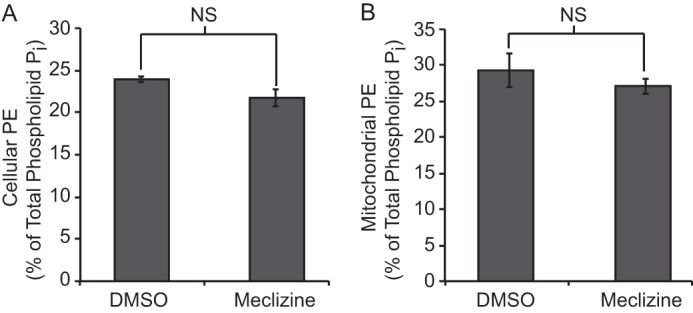
**Total cellular and mitochondrial phosphatidylethanolamine levels in meclizine-treated fibroblasts.** MCH58 fibroblasts were grown in the presence of meclizine (50 μm) or DMSO for 5 h followed by cellular (*A*) and mitochondrial phospholipid (*B*) extraction as described under “Experimental Procedures.” Phospholipids were separated using one-dimensional TLC, and phosphorous was quantified using the method of Bartlett (18). Data are expressed as the percentage of PE relative to total phospholipids and represent the average of three independent experiments (*NS*, not significant). *Error bars* represent S.D.

Recently, it has been shown that a modest reduction in PE in mammalian mitochondria impairs oxidative phosphorylation, reducing respiration (24). Therefore, we asked whether short term (5-h) inhibition of the Kennedy pathway of PE synthesis by meclizine causes a decrease in cellular and mitochondrial PE levels and contributes to reduced respiration. As shown in Fig. 6, *A* and *B*, we did not observe a significant decrease in the cellular or mitochondrial PE levels of human fibroblasts treated with meclizine, ruling out a reduction in PE as a cause for decreased respiration *in vivo*.

##### Inhibition of Respiratory Growth by Meclizine Is Accentuated through Addition of Ethanolamine

**FIGURE 7.**
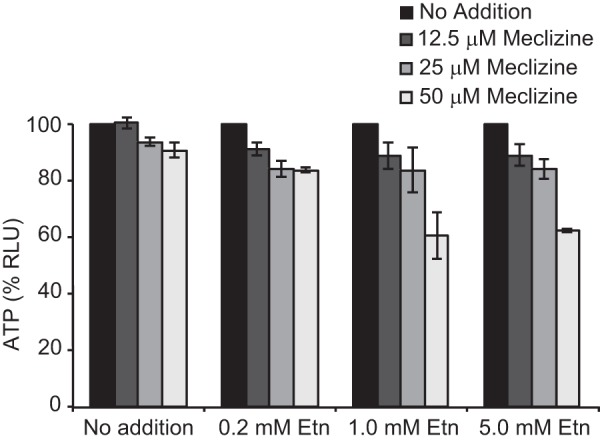
**Meclizine-mediated respiratory growth inhibition of human skin fibroblasts is enhanced by exogenous addition of ethanolamine.** MCH58 cells cultured in 10 mm galactose medium were exposed to different concentrations of meclizine and Etn for 24 h followed by the measurement of cellular ATP levels. Data were normalized to 100% for no treatment controls, and *errors bars* represent S.D. from mean (*n* = 3). *RLU*, relative luminescence units.

We originally identified meclizine as an agent that inhibits the growth of human skin fibroblasts grown in galactose *versus* glucose. It has long been known that when human cells are grown in galactose they are highly reliant on mitochondrial respiration. In the current study, we showed that in the presence of meclizine the phospholipid biosynthetic intermediate PEtn accumulated and directly inhibited mitochondrial respiration. Our model predicts that exogenous Etn should have an additive or synergistic effect with meclizine in inhibiting respiratory growth. Consistent with our model, we found that a combination of meclizine treatment with Etn supplementation synergistically reduced cellular ATP levels (Fig. 7).

## DISCUSSION

The current study provides the molecular basis for inhibition of respiration by meclizine, an over-the-counter antinausea and antivertigo drug. Using a combination of mass spectrometry, metabolic labeling, and *in vitro* biochemical assays, we found that PCYT2 is a direct target of meclizine. Our study highlights the use of metabolic profiling in deciphering mechanisms of drug action with important biological and clinical implications.

To our knowledge, this study is the first to pharmacologically link the Kennedy pathway of PE biosynthesis to mitochondrial energy metabolism and is consistent with a previous report showing that mice heterozygous for the *Pcyt2* gene have reduced energy production from fatty acid oxidation (25). PE is an essential phospholipid present in eukaryotic membranes and is highly enriched in mitochondrial membranes. It has overlapping functions with cardiolipin (26), a mitochondrion-specific phospholipid that is essential for optimal respiration (27). PE is synthesized by multiple biochemical pathways (21, 22), including the phosphatidylserine decarboxylase-catalyzed mitochondrial pathway and the cytosolic/endoplasmic reticulum CDP-Etn Kennedy pathway. The mitochondrial pathway contributes the bulk of mitochondrial PE that is retained in this organelle and contributes to mitochondrial function (28, 29). Recently, it has been shown that a decrease in mitochondrial PE by deletion of phosphatidylserine decarboxylase in the yeast *Saccharomyces cerevisiae* results in reduced respiration (30). A similar reduction in mitochondrial respiration has been observed in mammalian cells where mitochondrial phosphatidylserine decarboxylase is depleted (24). However, the non-mitochondrial CDP-Etn Kennedy pathway of PE synthesis has never been linked to a respiratory defect in either yeast or mammalian cells.

How does direct inhibition of PCYT2 lead to attenuation of mitochondrial respiration? Two possibilities exist. 1) The accumulation of upstream metabolites (PEtn) could interfere with mitochondrial respiration, or 2) depletion of downstream metabolites (PE) could alter mitochondrial membrane structure, thereby inhibiting respiration. We favor the first hypothesis, which is supported by our observation that the intracellular concentration of PEtn in meclizine-teated fibroblasts increased to a level sufficient to inhibit mitochondrial respiration (Figs. 1*D* and 5, *A* and *B*). Notably, our *in vitro* data on PEtn inhibition of mitochondrial respiration is consistent with a previous study that showed that both Etn and PEtn inhibit respiration in isolated mitochondria (23). The second mechanism seems less likely because the bulk of mitochondrial PE is synthesized *in situ* by the action of phosphatidylserine decarboxylase. Moreover, we did not observe any decrease in cellular or mitochondrial PE of the meclizine-treated fibroblasts, discounting the second possibility (Fig. 6). The synthetic interaction between meclizine and Etn in MCH58 cells further buttresses the first model because addition of Etn to meclizine-treated cells exacerbated, rather than alleviated, the cell viability as measured by ATP levels (Fig. 7).

According to our model, meclizine itself has no effect on mitochondria but rather blocks PCYT2, leading to the accumulation of PEtn, which is itself an endogenous inhibitor of respiration. Although multiple lines of evidence support our model, questions still remain. First, the genetic depletion of *PCYT2* by RNAi did not completely phenocopy the effect of meclizine on mitochondrial respiration, although this could be due to incomplete knockdown and the lack of sustained accumulation of PEtn (Fig. 4*E*). The inability of genetic silencing to fully phenocopy drug treatment is not uncommon (31) and in principle could be due to the presence of residual enzyme activity of PCYT2. Second, overexpression of *PCYT2* did not confer resistance to the effect of meclizine on respiration (data not shown), which could be due to tight regulation of its intracellular levels. Given that meclizine is known to target multiple cellular proteins (6–8), we cannot exclude additional mechanisms that may underlie its impact on respiration.

Regardless, we have clearly shown that meclizine inhibits PCYT2 and causes an increase in cytosolic PEtn to a level sufficient to inhibit mitochondrial respiration. To our knowledge, such a mechanism of respiratory inhibition has never been described before. The mechanism of PEtn-mediated inhibition of respiration appears to be distinct from canonical inhibitors of respiration, including inhibitors of electron transport (rotenone and antimycin), uncouplers (carbonyl cyanide 3-chlorophenylhydrazone and dinitrophenol), and ATP synthesis (oligomycin). PEtn addition to mitochondria resulted in reduced oxygen consumption, diminished membrane potential, and a decrease in NADH levels (Fig. 5), whereas treatment with rotenone or antimycin would have increased NADH levels, carbonyl cyanide 3-chlorophenylhydrazone would have increased oxygen consumption, and oligomycin would have increased membrane potential. The inhibition does not appear to be substrate-specific as we observed inhibition of respiration using complex I- or complex II-linked substrates. The precise mechanism by which accumulation of cytosolic PEtn inhibits respiration is currently not known, but our bioenergetics measurements suggest a mechanism whereby PEtn may interfere with the generation of reducing equivalents that feed into the respiratory chain.

Importantly, our work identifies for the first time an inhibitor of the CDP-Etn Kennedy pathway with therapeutic potential. This pathway has been implicated in a range of human disorders, including cancer (32) and ischemia-reperfusion injury of brain and heart (33), and infectious disorders, including African sleeping sickness and malaria (34, 35). Our own previous work has shown that meclizine, through blunting of respiration, is cytoprotective against ischemic injury to the brain and the heart (4) as well as polyglutamine toxicity observed in Huntington disease (5). Currently, effective therapies are not available for these disorders, making meclizine an attractive drug for repurposing. An intriguing question is to what extent the antivertigo and antinausea effects of meclizine may be occurring through targeting of PCYT2. A recent pharmacokinetics study on human subjects given an oral dose of 25 mg showed a peak plasma concentration of 80 ng/ml (∼0.2 μm) (36), which is almost 70-fold below the minimum concentration required for inhibition of respiration in cell lines we have tested (4); thus, currently approved doses are unlikely to be active on mitochondrial respiration. We anticipate that identification of PCYT2 as a direct molecular target of meclizine may help to guide its clinical development for new uses. It is notable that PEtn, which accumulates following inhibition, can be detected noninvasively using ^31^P NMR, providing a facile marker of pharmacodynamics.

## Footnotes

5 The abbreviations used are:

PEtn: phosphoethanolamine
PE: phosphatidylethanolamine
PCho: phosphocholine
PCYT2: CTP:phosphoethanolamine cytidylyltransferase
Etn: ethanolamine
OCR: oxygen consumption rate
ECAR: extracellular acidification rate.
